# Portal Hypertension as Immune Mediate Disease

**DOI:** 10.5812/hepatmon.18625

**Published:** 2014-06-07

**Authors:** Sara Manti, Lucia Marseglia, Gabriella D'Angelo, Martina Filippelli, Caterina Cuppari, Eloisa Gitto, Claudio Romano, Teresa Arrigo, Carmelo Salpietro

**Affiliations:** 1 Department of Pediatric Sciences, Genetics and Pediatric Immunology Unit, University of Messina, Messina, Italy

**Keywords:** Portal Hypertension, Children, Immune System, Endocrine System, Liver Fibrosis

## Abstract

**Context:**

Portal Hypertension (PH) is a progressive complication due to chronic liver disease. In addition to pathophysiologic changes in the micro-circulation, in PH are established fibrous tissue (periportal fibrous septal) and regenerative hyperplastic nodules (from micro- to macro-nodules) promoting hepatic architectural distortion.

**Evidence Acquisition:**

A literature search of electronic databases was undertaken for the major studies published from 1981 to today. The databases searched were: PubMed, EMBASE, Orphanet, Midline and Cochrane Library. We used the keywords: "portal hypertension, children, immune system, endocrine system, liver fibrosis".

**Results:**

It is believed that PH results from three “phenotype”: ischemia-reperfusion, involving nervous system (NS); edema and oxidative damage, involving immune system; inflammation and angiogenesis, involving endocrine system. However, its exact cause still underdiagnosed and unknown.

**Conclusions:**

PH is a dynamic and potentially reversible process. Researchers have tried to demonstrate mechanisms underlying PH and its related-complications. This review focuses on the current knowledge regarding the pathogenesis, and immune, endocrine-metabolic factors of disease. The strong positive association between immune system and development of PH could be efficient to identify non-invasive markers of disease, to modify prognosis of PH, and to development and application of specific and individual anti-inflammatory therapy.

## 1. Context

Portal hypertension (PH) occurs when portal pressure gradient or the pressure difference between the portal and inferior cava vein strongly increases. In the best of our knowledge, exact epidemiological data of PH are still unknown. However, its incidence is very similar to PH- related complication (ascites, hepatorenal syndrome, life threatening gastroesophageal bleeding, portosystemic encephalopathy, hepatopulmonary syndrome, hyperkinetic syndrome and sepsis). In fact, PH is diagnosed when its clinical manifestations appear (1). In healthy subjects, the portal venous pressure ranges from 7 to 10 mmHg and the hepatic venous pressure gradient changes from 1 to 4 mmHg. PH is clinically significant when portal vein pressures or portal vein to hepatic vein greater than 5 mmHg and 10 mm Hg gradient respectively (2). In adults, pressure gradients above 10 mmHg cause esophageal varices formation whether greater than 12 mmHg lead to ascites and variceal bleeding (3). On other hand, several children can present misunderstood presinusoidal PH. Therefore the diagnosis of PH often can be made by the indirect clinical signs as well as esophageal varices and/or splenomegaly. PH is a progressive complication due to chronic liver disease in which are established fibrous tissue (periportal fibrous septal) and regenerative hyperplastic nodules (from micro- to macro-nodules) promoting hepatic architectural distortion (4). Persistent hepatic structural alterations induce vasoconstriction which can dramatically increase local resistance to blood flow range from 20% to 30% (5). In addition to pathophysiologic changes in the micro-circulation of the liver, it has been also describe abnormalities of sinusoidal endothelial cells (SEC) (obstruction of endothelial fenestrae, accumulation of collagen within the space of Disse, development of a sinusoidal basement membrane) producing lower amounts of nitric oxide (NO) (3). This latter further favour an increase in vascular resistance.Adaptation to these consequences causes development of porto systemic collaterals, splanchnic arteriolar vasodilatation and splenomegaly (5). PH also persists for increased cardiac output (result from elevated venous return and decreased afterload). In conclusion, both, increased intra- and extra-hepatic resistance, lead to development and perpetuation of PH. However, these hemodynamic events have not been confirmed in pediatric patients. Therefore, in this population underlynig mechanisms are still unclear (Table 1) (1, 3).

**Table 1.** Etiology of Portal Hypertension in Children

Types of Portal Hypertension	Etiology
**Pre-hepatic**	Arteriovenous fistula
	Splenomegaly
	Congenital stenosis of the portal vein
	Portal vein thrombosis
**Intra-hepatic**	Autoimmune hepatitis
	Hepatitis B and C
	Idiopatic portal hypertension
	Gaucher’s disease
	Schistosomiasis
	Veno-occlusive disease
	Alfa 1 anti-trypsin deficiency
	Wilson’s disease
	Steato-hepatitis
	Glycosen storage disease type IV
	Toxins
	Biliary atresia
	Primary sclerosing cholangitis
	Primary biliary cirrhosis
	Caroll’s disease
	Congeital hepatic fibrosis
	Cystic fibrosis
	Peliosishepatis
	Familial cholestasis
	Choledochal cyst
**Post-hepatic**	Inferior vena cava obstruction
	Budd-Chiari syndrome
	Congestive heart failure

## 2. Evidence Acquisition

A literature search of electronic databases was undertaken for the major studies published from 1981 to today. The databases searched were: PubMed, EMBASE, Orphanet, Midline and Cochrane Library. We used the keywords: "portal hypertension, children, immune system, endocrine system, and liver fibrosis".

## 3. Results

### 3.1. Portal Hypertension and Nervous- Immune-Endocrine Systems: A Possible Interaction

Today, the pathogenetic mechanism of PH has not been fully understood. It is believed that PH results from three “phenotype”: ischemia-reperfusion, involving nervous system (NS); edema and oxidative damage, involving immune system; inflammation and angiogenesis, involving endocrine system (6). A large number of studies were conducted on role of NS in PH. Both in humans and animals, the mesenteric vascular system has sympathetic innervation, mediated by post-synaptic α1-adrenoreceptors (7). PH patients show a neural dysregulation. The signals of PH are detected via afferent nerves and transmitted to nucleus of the solitary tract, paraventricular nucleus and supraoptic nucleus. Efferent nerves originate from these nuclei. Blockade of any part of the reflex arc arrests development of vasodilation and hyperdynamic circulation in PH. Furthermore, PH is characterized by downregulation of mRNA and proteins involved in adrenergic transmission in the superior mesenteric artery (SMA) and sympathetic nerve atrophy/regression in the mesenteric arterial vasculature. Therefore, this mechanism might contribute to aggravating splanchnic vasodilation associated with PH (8). Otherwise, Bockx et al. demonstrated that vagus nerve stimulation improves portal hypertension. Vagus nerve also acts on liver. Its efferent neurotransmitters are acetylcholine and vasoactive intestinal peptide are vasodilators. Therefore it improves portal hypentesion (9). Moreover, taking into consideration the biological activity of immune system, it seems likely that a significant role in the development of pathological changes in the liver is played byan immunological imbalance. PH often appears with other autoimmune diseases such as systemic lupus erythematosus, systemic sclerosis, Raynaud’s phenomenon, celiac disease, and chronic thyroiditis (10). Several hypothesis have been given to explain these associations. Moreover, in patients affected by PH it was detected the presence of anticardiolipin antibody and anti-RNP antibody (11). Probably, immunoglobulins (Ig) an interference with prostacyclinsynthesis and favours obliteration of small vessels portal and hepatic veins (12). It has been also proposed that the alterated clearance of the circulating immune complexes by hepatic kupffer cells (KC) may further favour abnormal deposition and trombosis. This leads to focal ischemia, therefore remaining well-perfused areas induce neo-angiogenesis (capillarisation of the sinusoids), increse shunt formation and subversion of liver parenchyma (13). On other hand, in patients with primary hypogammaglobulinemia was also demonstrated severe histological features, especially rareliver disorders such as nodular regenerative hyperplasia (14). PH is strongly correlated to liver injury and fibrosis, and, pathogenically, to several inflammatory pathways. Immune system also influences migration and proliferation of fibroblasts and deposition of connective tissue. Precisely, the hepatic architectural distortion could be mediated by elevated serum levels of Transforming Growth Factor (TGF)-β and connective tissue growth factor (15). Buck et al. demonstrated that hepatic vein pressure gradient is significantly influenced by inflammatory biomarkers such as Fas, interleukin (IL)-1Ra, IL-1ß, and VCAM-1 (16). Although, experimental studies have demonstrated that Fas (sFas)/Fas-ligand (FasL) signalling system plays a key role in liver failure, its role is not yet known. Probably, it can positively interfere with apoptosis induction (17). VCAM-1 is an adhesion molecule expressed in the sinusoidal and portal endothelial cells. It favours the interaction between lymphocytes and hepatic cells. It has been detected incresead serum VCAM-1 levels in PH (18). IL-1, produced by activated macrophages inside the spleen, also plays a link role between portal vein pressure and PH- related complications. It seems that IL-1ß up-regulates expression of NALP-3, family member NLRP3 (nucleotide binding domain, leucine rich repeats-containing), enhancing inflammatory responses (19). In rats affected by PH, it has been noted an increase in hepatic release of IL-1ß associated with fatty infiltration in mitochondria. This morphologic mitochondrial alteration, also called megamithocondria, could be involved in the etiopathogenesis of PH (20).

Serum IL-6 levels also influencethe degree of liver failure. In addition to elevated blood nitric oxide (NO) levels, IL-6 correlates with portal-blood flow, hepatic congestion, and possible dilation of oesophageal veins (21). Tumor necrosis factor alpha (-a), TNF receptor-I and TNF receptor-II might act by the same mechanisms. TNF receptor-I/II would seem to have a predictive potential role in surgery-treated patients with PH (22). TNF-a promotes the NO release (23). KCs- derived TNF-a is mitogenic and chemoattractant for HSC (24). However, the nature of these data is still controversial (25). Tokushige et al. reported a significant increase of serum TNF-a levels and altereted Th1/Th2 balance (26). It has been reported a decrease of serum Th2 levels, suggesting that this mechanisms coul be associated with the pathogenesis of PH. In fact, studies demonstrated that activated KCs might lead to apoptosis in CD95^+^ T lymphocytes and hepatocytes (27). KCs are acting as antigen presenting cells, recruit CD8^+^ and regulatory T cells. Adhesivemolecules (such as VCAM and ICAM-1) allow KCs to maintain contact with lymphocyte. By direct contact, T cells are driven to apoptosis (Table 2) (24, 28). Although lymphocyte density is decreased, especially in the spleen, the total amount of lymphocytes is increased for hypersplenism due to PH (29). Another possible reason is that long-term contact between exogenous molecules and environment splenic promotes and enhances lymphocyte response (24).

**Table 2.** Distribution of Intra-Hepatic T Lymphocytes^a^

T-Cell- Phenotype	Liver
**CD4**	22
**CD8**	72
**CD4^+^**	5
**CD8^+^**	5
**CD4^-^**	14
**CD8^-^**	5
**CD8a^+^ß^-^**	15.4
**CD56**	32

^a^ Data are presented as %.

Ziol et al. demonstrated a high percentage of CD8^+^/CD3^+^/CD57^+^ cytotoxic T cells in liver sinusoids. Therefore, they hypothesized that lymphocyte could participate in the pathogenesis of nodular regenerative hyperplasia (NRH). T cells promote NRH by several ways: they are strongly expressing granzyme B, responsible for endothelial injury; they are recruited to the liver and located in atrophic areas; they also are able to achieve antigen (intra- and/or extra-hepatic) recognitionand cytotoxicity in a non–major histocompatibilitycomplex (MHC) (30). Moreover, Guo and co-workers reported that T lymphocyte subsets (CD4^+^CD25^+^CD127 low/-Treg) and Foxp3 ratio was strongly increased in subjects affected by hypersplenism and PH (31). In addition, reduced antigen-presenting ability of non-T cells might further promote immunological dysregulation. Merino et al. reported that these inflammatory alterations could be also drive by chemotactic cytokines (fractalkine or CXC3CL1 and stromal cell-derived factor alpha or SDF1-a) and their respective receptors (CXC3CR1 and CXCR4). Chemokines are differentially expressed during chronic liver diseases (32).

Therefore, in the absence of an adequate immune defense, gut-bacteria and/or bacterial-derived antigens, also known pathogen-associated molecular patterns (PAMPs), can more easily reach portal venous system, promoting fibrosis and PH, recruiment of inflammatory extra-hepatic and hepatic cells, and acting as “cytokine-releasing” organ (32). IL-10 is a pleiotropic and anti-inflammatory cytokine. Gut flora of patients with PH can produce, trough T and B lymphocytes, monocytes/macrophages, mast-cells, endotoxin, glucocorticoids, reactive oxygen intermediates, and pro-inflammatory cytokines (such as TNF-αand IL-1), IL-10. Bacteria colonizing the gut are also capable of inducing production, it plays an important role as a chemotactic factor, activating eosinophiles, basophiles, and neutrophiles, and T lymphocytes and drawing them to the place where a toxic agent is working. IL-8 does not seem to play a role in the hyperdynamic circulation. All these phenomenaare also known as “leaky gut syndrome”, it is characterized by increased gut permeability, bacterial overgrowth, and changes in the composition of gut-flora. These mechanisms lead to systemic complication (33). On other hand, monocyte and neutrophil recruitment, through KCs derived IL-6, IL-12, IL-1β, TNF-α, NO and chemokines (MIP-1α/β, MCP-1, MIP-2) limits the infection (34). Disease fibrogenic processes are further induced by lipopolysaccharide (LPS) bacterial ligand of Toll-like receptor (TLR) and incresead serum leptin levels, especially in patients affected by hepatitis C virus (Table 3) (35, 36).

**Table 3.** Hepatic Cells and Their Specific Toll Like Receptor ^a^

Cell Type	TLR- Expression
**Hepatocyte**	TLR 1-9
**Kupffer cell**	TLR 2,4
**Stellate cell**	TLR 2-4
**Sinusoidal endothelial cell**	TLR 4

^a^ Abbreviation: TLR, Toll Like Receptor.

TLRs, a family of transmembrane-protein receptors, recognize bacteria, fungi, and virus and play a critical role in the induction of innate immune responses through inflammatory cytokine including IFN. Especially, TLR4 over-expression confers hypersensitivity to LPS and higher release of vasoconstrictor molecules after endotoxin-induced KC activation. Previous studies also showed that the TLR4/ liver endothelial cells pathway, by effector protein MyD88, also regulates liver TGF-β-mediated fibrosis, and angiogenesis (37). On other hand, the splenic expression of TLR4 might be a further cause of PH due to hypersplenism. Probably, bacterial overgrowthenhances expression of TLR4 on splenic macrophage that destroy red blood cells.TLR2 genetic variants favours alterated intestinal permeability and elevated risk of bacterial translocation (38).

PH also results from an increase local resistance to blood flow. The intact endothelium has a crucial role in vascular tone, as main source of vasoconstrictor and vasodilator molecules (25). In PH, the massive release of vasoconstrictors, derived from arachidonic acid, such as thromboxane (TX)-A2 or cysteinyl leukotrienes (Cys-LTs, leukotrienes C4, D4, E4), and decreased synthesis of vasodilators further promote a vascular hyper-tone (39). In addition, it has been also demonstrated vasoconstrictor role of endothelin (ET)-1, ET-3, promoting increased intrahepatic resistances. Cytokines, epinephrine, vasopressin, and angiotensin-II induce the synthesis of ET-1 and ET-3. These are acting through a specific receptor, named A and B, on smooth muscle cells, HSCs, endothelial and sinusoidal endothelial cells. Activation of ET-A receptor induces vasoconstriction while activation of ET-B leads to vasodilation. Serum ET-1/e levels are positively correlated with degree of PH (40, 41). Vascular remodeling processes are also resulting from “hyperdynamic circulation” and/or “forward flow” theory (42). This has been observed in all forms of PH. It is due to the presenceof both increased splanchnic blood flow and higher portal vascular resistance (43). Several are involved molecules. NO, arterial vasodilatator factor acting through guanylyl cyclase, is producted by endothelial nitric oxide synthases (eNOS), in the splanchnic arterial circulation; by neuronal NOS (nNOS), in the nervous system; by mitochondrial nitric oxide synthase (mNOS); and by inducibleNOS (iNOS), in several cell types such as vascular smooth muscle cells and macrophages. NO exerts a paradoxical role. Altered inflammatory response in patients with PH, promotes the production of NO, enhancing cyclic guanosine 3’-5’-monophosphate (cGMP) related- hyperdynamic circulatory syndrome.On other hand, NO deficiency leads to elevated vascular resistances (23, 44). eNOS related-NO can be reduced by endogenous circulating amino acid asymmetric dimethylarginine (ADMA). ADMA, synthesized by proteolysis of citrulline and dimethylamine, is associated with multiorgan failure, especially liver damage (45).

In addition to heme oxygenase, guanylyl cyclasealso induces the release of carbon monoxide (CO), endogenous regulator that further contributes, in the early stage of PH to maintan arterial vasodilatation and systemic hyperdynamic circulation. In fact, CO is required for maintaining hepatic microvascular blood flow. CO also acts as a potent anti-inflammatory molecule that reduces synthesis of the pro- inflammatory cytokines (such as TNF-α, IL-1βand MIP-1β (46). Otherwise, endothelium-derived hyperpolarizing factor (EDHF) seems especially to act in arterioles and smaller arteries (47). The vasodilators also involved includeglucagon, prostacyclin (PGI2), endocannabinoids, adrenomedullin and hydrogen sulfide (H2S) (42). It is a recently discovered gas neurotransmitter, generated through a trans-sulfuration pathway. Its putative roleis still unexplored. Several studies suggest that H2S can also reduce systemic blood pressure (48). Interferon (IFN)-y exerts similar effects on microvasculature of portal tracts. It is a Th1 cytokine that inhibits stellate cell proliferation, fibrogenesis, and muscle-specific gene expression. Its action is mediated by HLA-DR antigen which, highly expressed on microvessels, is involved in immune recognition. It remains doubtful whether IFNγ exercises an inhibitory effect on HSC through pre-pro-endothelin (ET)-1 inhibition (18).

### 3.1. Endocrine-Inflammatory Response and PH

These events lead to activation of and extra-hepatic and hepatic cells, promoting fibrosis and PH. KCs, also known as hepatic macrophages, are activated. Marker of KCs activation is CD163, a macrophage lineage-specific haemoglobin-haptoglobin scavenger receptor induced by TNF-a. CD163 also predict both degree of PH and liver dysfunction (49). KCs are the main source of inflammatory (e.g. eicosanoids, chemokines, superoxide nitric oxide, proteolytic and lysosomal enzymes, NO, TNF-a, and IL-6) and/or anti-inflammatory (e.g. detoxifying agents, glutathione, IL-10, IL-18) molecules. In fact, KCs are both involved in the progression and regression of liver damage (50). KC can act in two ways: release vasoactive molecules and enhance and maintain the transformation of quiescent hepatic stellate cells (HSCs) towards so-called myofibroblast-like cells, eliciting fibrosis through synthesis of extracellular matrix, increased vascular distortion and resistance, thereby promoting portal hypertension (13). The conversion involves the loss of vitamina A and lipid store, expression of contractile and migratory properties favored by alpha-smooth muscle actin (a-SMA), increased half-life cellular mediated by transcription factor nuclear factor-kB (NF-kB), and transcriptional repressor activation such as Rev-erb-alpha (51). This has recently been discovered, its role is not still clear. Probably, it promotes both pro- or anti-fibrogenic response and a contractile phenotype of HSCs (52).

HSCs contraction can be also mediated by CXCL12 in CXCR4-specific manner, through a calcium-independent pathway (53). In addition to CXCL12-CXCR4 pathway, Rho-kinase activation also enhances cell contraction, intrahepatic resistance and consequently portal pressure (54). Sinusoidal endothelial cells (SEC) are directly and/or indirectly partecipate to liver fibrosis and angiogenesis.This phenomenon is also called “endothelial-mesenchymal transition”. Precisally, HSC and SEC release growth factors that influence the critical role of one and the other. The link between angiogenesis and fibrosis is hypoxia. It promotes the release of several angiogenic growth factorsvascular such as endothelial growth factor (VEGF); platelet-derived growth factor (PDGF); hypoxia inducible factor 1 alpha (HIF-a), an oxigen sensitive transcription factor; and angiopoietin-1, inducing capillarization and collagen deposits in perisinusoidal space (55). VEGF contributes to elevated overall blood flow in spleen. VEGF exercits its action trough a VEGF-eNOS-phosphatidylinositol-3-kinase (PI3K-Akt) pathway. Precisely, VEGF stimulates eNOS and PI3K-Akt which in turns increasing release of NO. Therefore, this axis is involved in the maintenance of hyperdynamic circulation in portal hypertension (56). Furthermore, VEGF, activating proliferation of endothelial tubule and cells, promotes formation of new portal-systemic collateral vessels (57). Otherwise, PDGF pathway modulates maturation of new vessels. Angiopoietin-1, a member of the Ang family, acts by binding to Tie2, a tyrosine kinase receptor expressed on liver vessels. It is involved in survival and recruitment endothelial cells. It is also been reported increased angiopoietin-1 levels in patients with PH (58). Cytokines that alsoorchestrate fibrosis are IFN-a; TNF-a; TGF-ß, activated by avß6 integrin,contributes to apoptotic activation through tBcl-2 and caspase family of protein (59); PDGF (57); angiotensin II; cannabinoid receptor CB1/CB2 signaling (60); and HMG-CoA-reduktase, it can enhance eNOS expression, vasodilatation and formation of portal-systemic collaterals (61-63).

The RAS (renin-angiotensin system) is recognized as an important regulator of portal pressure. Liver damage induces activaton of RAS, angiotensin-converting enzyme and their specific receptors which promote inflammation, synthesis of collagene and PH. In fact, angiotensin II, by binding angiotensin type I receptors, promotes profibrogenic effect mediated by TGF-ß1. Apelin also binds angiotensin-like- receptor 1. Several studies were conducted on critical role of apelin. It is endogenous ligand; it is expressed on nervous, cardiac, gastrointestinal, and hepatic cells. Precisely, it is over-expressed in HSCs where it promotes collateral circulation. Chen and co-workers proposed apelin as a new possible prognostic factor in PH patients (64). KC-derived TGF-ß seems to partecipate to transformation of HSC, promoting release of proteoglycans and collagen, and induce mRNA expression of metalloproteinases (MMPs) (65). Fibrogenic factors also include reactive oxygen species, antiapoptotic proteins, and tissue inhibitors of matrix metalloproteinases (TIMPs). These latter inhibit MMPs, proteolytic enzymes containing metal ions, that degrade all types of ECM, such as collagen (I, IV, V, VII, X, XI), fibronectin, hyaluronan, undulin, elastin, and proteoglycans, which are then released into the circulation. Therefore, it has been hypothesized that all small fragments of degraded extracellular matrix can be used as markers reﬂect the severity of disease (66). MMPs activation is probably mediated by heparin- and chitin-binding glycoprotein (YKL-40). YKL-40, expressed on the surface of spleen, acts as growth and migration factor in muscle and connective tissue, This further confirms that portal hypertension and splenomegaly influence each other (67). Cannabinoids are a group of molecules binding CB1 and CB2 receptors on nervous, liver and gut cells, and adipocytes. In normal liver their expression is absent or low. During chronic progressive liver diseases, their serum levels are increasing and promoting fibrosis in early stage. Otherwise, cannobinoids contribute to splanchnic vasodilatation and PH in end-stage liver disease (68).

Moreover, genetic factors have been recognized as playing an important role in the development of PH. Single-nucleotide polymorphisms (SNPs) that influence hepatic architectural distortion are: TNF-a, IL-10, TGF-ß, CCR5, angiotensinogen, and peroxisome proliferator-activated receptor α (PPARα). It, transcription factor activated by ligands, influences genes related to PH pathway such as oxidative stress, vascular tone, and fibrogenesis (69). However, othermechanisms promoting PH are still unclear. PH seems to be also endocrine- metabolic-immunological changes. Hormone molecules also appear to influence fibrosis. Recently, it has been demonstrated that elevated serum levels of insulin, leptin, and adiponectin.

Insuline resitance (IR) is strongly associated with the progression of chronic liver disease. Although molecular pathways explaining the relationship between insulin resistance and PH are largely unknown, it has been hypothesized thatIR can resultfrom two ways: reduced insulin degradation due to liver failure, and development of intra- and extra-hepatic collateral circulation, contributing to decreased insulin clearance (70, 71). IR can cause sinusoidal endothelial dysfunction, decrease in NO production, and increment of peripheral vascular resistance. Leptin, trough specific receptors (ObR) in HSCs, might upregulate several signaling pathways involved in angiogenic and fibrotic mechanisms. In fact, leptin induce synthesis of collagen; release of VEGF, angiopoietin-1 and HIF-a; monocyte chemoattractant protein 1 (MCP-1) and NF-kB expression (72-75). However, in humans the crtical role of leptin is still unclear. Delgado et al. demonstrated that blockade of leptin- NO pathway is significantly associated with reduction of PH (76). Adiponectin may influence liver fibrosis. In fact, inducing IL-10 release by KCs, also inhibits TNF-α synthesis. In addition, elevated serum adiponectin levels inhibit HSCs proliferation, favouring cellular apoptosis and inducing HSCs quiescence. Otherwise, in the late stage of PH, it was noted normal or reduced serum adiponectin levels. A less hepatic extraction is the most common cause of this phenomenon. In addition to serum insuline levels, adiponectin significantly predict the presence of PH related-complications such as esophageal varice and bleeding risk (77). Recently, it was discovered helpful effects of the peptide hormone relaxin. It induces, by binding with specific receptor (RXFP1) expressed on HSCs, reduction of contractile filament expression, PH and collagen deposition. It has been demonstrated that relaxin is associated with increased expression of the fibrillar collagen-degrading enzyme MMP13, decreased expression of TIMP2, and impaired TGFβ signalling.However, this function has only been demonstrated in pathologically distinct PHT models. In fact, in early cirrhosis relaxin seems to promote portal blood flow. Moreover, relaxin reverses insulin resistance. It increases vascular reactivity and angiogenesis which in turns favours an increase in peripheral glucose utilization and in muscle glucose uptake (78).

## 4. Conclusions

PH is a dynamic and potentially reversibleprocess. Researchers have tried to demonstrate mechanisms underlying PH and its related-complications. Several studies showed that PH continuously varies according to degree and duration of injury, angiogenesis, and areas of fibrosis. The influence of the immune system on the development of PH has recently been the object of attention. However, the connection between PH, altered immune response, and development of changes in the liver has not been fully explained. Here, we want highlight the critical role of immunological disregulation underlying all stages of the disease. The strong positive association between immune system and development of PH could be efficient to identify patients with increased hepatic vein pressure gradient and to modify prognosis of PH, especially unricognized form of liver disease. Furthermore, non-invasive markers could emerge as an alternative to the staging of severity disease by means of invasive procedures. Longitudinal studies have already shown their utility as predictors of complications from portal hypertension and mortality. It would be very attractive for daily clinical practice. Moreover, a better knowledge of the immunological pathogenic mechanisms might be useful to development and application of specific and individual anti-inflammatory therapy. Further investigations are also required to understand the relationship of these processes.

## References

[1] Imanieh MH, Dehghani SM, Khoshkhui M, Malekpour A (2012). Etiology of Portal Hypertension in Children: A Single Center’s Experiences.. Middle East J Dig Dis ..

[2] Vargas HE, Gerber D, Abu-Elmagd K (1999). Management of portal hypertension-related bleeding.. Surg Clin North Am..

[3] Gugig R, Rosenthal P (2012). Management of portal hypertension in children.. World J Gastroenterol..

[4] Garcia-Tsao G, Sanyal AJ, Grace ND, Carey WD, Practice Guidelines Committee of American Association for Study of Liver D, Practice Parameters Committee of American College of G (2007). Prevention and management of gastroesophageal varices and variceal hemorrhage in cirrhosis.. Am J Gastroenterol..

[5] Gupta TK, Toruner M, Chung MK, Groszmann RJ (1998). Endothelial dysfunction and decreased production of nitric oxide in the intrahepatic microcirculation of cirrhotic rats.. Hepatology..

[6] Aller MA, Arias JL, Cruz A, Arias J (2007). Inflammation: a way to understanding the evolution of portal hypertension.. Theor Biol Med Model..

[7] Piascik MT, Soltis EE, Piascik MM, Macmillan LB (1996). Alpha-adrenoceptors and vascular regulation: molecular, pharmacologic and clinical correlates.. Pharmacol Ther..

[8] Coll M, Martell M, Raurell I, Ezkurdia N, Cuenca S, Hernandez-Losa J (2010). Atrophy of mesenteric sympathetic innervation may contribute to splanchnic vasodilation in rat portal hypertension.. Liver Int..

[9] Bockx I, Verdrengh K, Vander Elst I, van Pelt J, Nevens F, Laleman W (2012). High-frequency vagus nerve stimulation improves portal hypertension in cirrhotic rats.. Gut..

[10] Valeria C, Lacquaniti A, Salpietro V, Nicoletta L, Ferrau V, Piraino B (2013). Thyroid dysfunction in thalassaemic patients: ferritin as a prognostic marker and combined iron chelators as an ideal therapy.. Eur J Endocrinol..

[11] Saito K, Nakanuma Y, Takegoshi K, Ohta G, Obata Y, Okuda K (1993). Non-specific immunological abnormalities and association of autoimmune diseases in idiopathic portal hypertension. A study by questionnaire.. Hepatogastroenterology..

[12] Carreras LO, Defreyn G, Machin SJ, Vermylen J, Deman R, Spitz B (1981). Arterial thrombosis, intrauterine death and "lupus" antiocoagulant: detection of immunoglobulin interfering with prostacyclin formation.. Lancet..

[13] Goldschmidt I, Baumann U (2012). Hepatic fibrosis in paediatric liver disease.. Clin Res Hepatol Gastroenterol..

[14] Malamut G, Ziol M, Suarez F, Beaugrand M, Viallard JF, Lascaux AS (2008). Nodular regenerative hyperplasia: the main liver disease in patients with primary hypogammaglobulinemia and hepatic abnormalities.. J Hepatol..

[15] Nakanuma Y, Sato Y, Kiktao A (2009). Pathology and pathogenesis of portal venopathy in idiopathic portal hypertension: Hints from systemic sclerosis.. Hepatol Res..

[16] Buck M, Garcia-Tsao G, Groszmann RJ, Stalling C, Grace ND, Burroughs AK (2014). Novel inflammatory biomarkers of portal pressure in compensated cirrhosis patients.. Hepatology..

[17] Elsing C, Harenberg S, Stremmel W, Herrmann T (2007). Serum levels of soluble Fas, nitric oxide and cytokines in acute decompensated cirrhotic patients.. World J Gastroenterol..

[18] Rizzoni D, Porteri E, Guefi D, Piccoli A, Castellano M, Pasini G (2000). Cellular hypertrophy in subcutaneous small arteries of patients with renovascular hypertension.. Hypertension..

[19] Xia Z, Wang G, Wan C, Liu T, Wang S, Wang B (2010). Expression of NALP3 in the spleen of mice with portal hypertension.. J Huazhong Univ Sci Technolog Med Sci..

[20] Prieto I, Jimenez F, Aller MA, Nava MP, Vara E, Garcia C (2005). Tumor necrosis factor-alpha, interleukin-1beta and nitric oxide: induction of liver megamitochondria in prehepatic portal hypertensive rats.. World J Surg..

[21] Mirodzhov GK, Avezov SA, Giiasov MM, Abdullaeva ZM (2012). [The role of interleukin-6 and nitric oxide in pathogenesis of portal hypertension and decompensation of liver cirrhosis].. Klin Med (Mosk)..

[22] Trebicka J, Krag A, Gansweid S, Schiedermaier P, Strunk HM, Fimmers R (2013). Soluble TNF-alpha-receptors I are prognostic markers in TIPS-treated patients with cirrhosis and portal hypertension.. PLoS One..

[23] Tazi KA, Moreau R, Herve P, Dauvergne A, Cazals-Hatem D, Bert F (2005). Norfloxacin reduces aortic NO synthases and proinflammatory cytokine up-regulation in cirrhotic rats: role of Akt signaling.. Gastroenterology..

[24] Withers DR, Kim MY, Bekiaris V, Rossi SW, Jenkinson WE, Gaspal F (2007). The role of lymphoid tissue inducer cells in splenic white pulp development.. Eur J Immunol..

[25] Francque S, Laleman W, Verbeke L, Van Steenkiste C, Casteleyn C, Kwanten W (2012). Increased intrahepatic resistance in severe steatosis: endothelial dysfunction, vasoconstrictor overproduction and altered microvascular architecture.. Lab Invest..

[26] Tokushige K, Yamauchi K, Komatsu T, Takasaki K, Hayashi N (2000). Predominant T helper 1 cells in patients with idiopathic portal hypertension.. J Gastroenterol Hepatol..

[27] Muschen M, Warskulat U, Peters-Regehr T, Bode JG, Kubitz R, Haussinger D (1999). Involvement of CD95 (Apo-1/Fas) ligand expressed by rat Kupffer cells in hepatic immunoregulation.. Gastroenterology..

[28] Lemaigre F, Zaret KS (2004). Liver development update: new embryo models, cell lineage control, and morphogenesis.. Curr Opin Genet Dev..

[29] Li ZF, Zhang S, Lv GB, Huang Y, Zhang W, Ren S (2008). Changes in count and function of splenic lymphocytes from patients with portal hypertension.. World J Gastroenterol..

[30] Ziol M, Poirel H, Kountchou GN, Boyer O, Mohand D, Mouthon L (2004). Intrasinusoidal cytotoxic CD8+ T cells in nodular regenerative hyperplasia of the liver.. Hum Pathol..

[31] Guo Y, Wu CZ, Liao Y, Zhang QY (2013). The expression and significance of CD4+CD25+CD127low/- regulatory T cells and Foxp3 in patients with portal hypertension and hypersplenism.. Hepatogastroenterology..

[32] Merino J, Aller MA, Rubio S, Arias N, Nava MP, Loscertales M (2011). Gut-brain chemokine changes in portal hypertensive rats.. Dig Dis Sci..

[33] Aller MA, de las Heras N, Nava MP, Regadera J, Arias J, Lahera V (2013). Splanchnic-aortic inflammatory axis in experimental portal hypertension.. World J Gastroenterol..

[34] Ebe Y, Hasegawa G, Takatsuka H, Umezu H, Mitsuyama M, Arakawa M (1999). The role of Kupffer cells and regulation of neutrophil migration into the liver by macrophage inflammatory protein-2 in primary listeriosis in mice.. Pathol Int..

[35] Salpietro C, Rigoli L, Miraglia Del Giudice M, Cuppari C, Di Bella C, Salpietro A (2011). TLR2 and TLR4 gene polymorphisms and atopic dermatitis in Italian children: a multicenter study.. Int J Immunopathol Pharmacol..

[36] McHutchison J, Goodman Z, Patel K, Makhlouf H, Rodriguez-Torres M, Shiffman M (2010). Farglitazar lacks antifibrotic activity in patients with chronic hepatitis C infection.. Gastroenterology..

[37] Zhu Q, Zou L, Jagavelu K, Simonetto DA, Huebert RC, Jiang ZD (2012). Intestinal decontamination inhibits TLR4 dependent fibronectin-mediated cross-talk between stellate cells and endothelial cells in liver fibrosis in mice.. J Hepatol..

[38] Li ZF, Zhang Y, Gao J, Zhang PJ, Wang JX, Liu XG (2004). [Expression and significance of Toll-like receptor 4 of splenic macrophage in patients with hypersplenism due to portal hypertension].. Zhonghua Yi Xue Za Zhi..

[39] Steib CJ, Bilzer M, op den Winkel M, Pfeiler S, Hartmann AC, Hennenberg M (2010). Treatment with the leukotriene inhibitor montelukast for 10 days attenuates portal hypertension in rat liver cirrhosis.. Hepatology..

[40] Cavasin MA, Semus H, Pitts K, Peng Y, Sandoval J, Chapo J (2010). Acute effects of endothelin receptor antagonists on hepatic hemodynamics of cirrhotic and noncirrhotic rats.. Can J Physiol Pharmacol..

[41] Gemelli M, Manganaro R, Mami C, Buemi M, Paolata A, Marrone T (2005). Endothelin-1 concentrations in cord blood of neonates with meconium-stained amniotic fluid.. J Perinat Med..

[42] Iwakiri Y, Groszmann RJ (2006). The hyperdynamic circulation of chronic liver diseases: from the patient to the molecule.. Hepatology..

[43] Vorobioff J, Bredfeldt JE, Groszmann RJ (1984). Increased blood flow through the portal system in cirrhotic rats.. Gastroenterology..

[44] Gitto E, Pellegrino S, Aversa S, Romeo C, Trimarchi G, Barberi I (2012). Oxidative stress and persistent pulmonary hypertension of the newborn treated with inhaled nitric oxide and different oxygen concentrations.. J Matern Fetal Neonatal Med..

[45] Snowdon VK, Guha N, Fallowfield JA (2012). Noninvasive evaluation of portal hypertension: emerging tools and techniques.. Int J Hepatol..

[46] Chen YC, Gines P, Yang J, Summer SN, Falk S, Russell NS (2004). Increased vascular heme oxygenase-1 expression contributes to arterial vasodilation in experimental cirrhosis in rats.. Hepatology..

[47] Iwakiri Y, Tsai MH, McCabe TJ, Gratton JP, Fulton D, Groszmann RJ (2002). Phosphorylation of eNOS initiates excessive NO production in early phases of portal hypertension.. Am J Physiol Heart Circ Physiol..

[48] Fiorucci S, Antonelli E, Mencarelli A, Orlandi S, Renga B, Rizzo G (2005). The third gas: H2S regulates perfusion pressure in both the isolated and perfused normal rat liver and in cirrhosis.. Hepatology..

[49] Gronbaek H, Sandahl TD, Mortensen C, Vilstrup H, Moller HJ, Moller S (2012). Soluble CD163, a marker of Kupffer cell activation, is related to portal hypertension in patients with liver cirrhosis.. Aliment Pharmacol Ther..

[50] Kolios G, Valatas V, Kouroumalis E (2006). Role of Kupffer cells in the pathogenesis of liver disease.. World J Gastroenterol..

[51] Oakley F, Meso M, Iredale JP, Green K, Marek CJ, Zhou X (2005). Inhibition of inhibitor of kappaB kinases stimulates hepatic stellate cell apoptosis and accelerated recovery from rat liver fibrosis.. Gastroenterology..

[52] Li T, Eheim AL, Klein S, Uschner FE, Smith AC, Brandon-Warner E (2014.). Novel role of nuclear receptor rev-erbalpha in hepatic stellate cell activation: Potential therapeutic target for liver injury.. Hepatology..

[53] Saiman Y, Agarwal R, Hickman DA, Fausther M, El-Shamy A, Dranoff JA (2013). CXCL12 induces hepatic stellate cell contraction through a calcium-independent pathway.. Am J Physiol Gastrointest Liver Physiol..

[54] Klein S, Van Beuge MM, Granzow M, Beljaars L, Schierwagen R, Kilic S (2012). HSC-specific inhibition of Rho-kinase reduces portal pressure in cirrhotic rats without major systemic effects.. J Hepatol..

[55] Lee S, Chen TT, Barber CL, Jordan MC, Murdock J, Desai S (2007). Autocrine VEGF signaling is required for vascular homeostasis.. Cell..

[56] Marques C, Licks F, Zattoni I, Borges B, de Souza LE, Marroni CA (2013). Antioxidant properties of glutamine and its role in VEGF-Akt pathways in portal hypertension gastropathy.. World J Gastroenterol..

[57] Fernandez M, Mejias M, Garcia-Pras E, Mendez R, Garcia-Pagan JC, Bosch J (2007). Reversal of portal hypertension and hyperdynamic splanchnic circulation by combined vascular endothelial growth factor and platelet-derived growth factor blockade in rats.. Hepatology..

[58] Ward NL, Haninec AL, Van Slyke P, Sled JG, Sturk C, Henkelman RM (2004). Angiopoietin-1 causes reversible degradation of the portal microcirculation in mice: implications for treatment of liver disease.. Am J Pathol..

[59] Popov Y, Patsenker E, Stickel F, Zaks J, Bhaskar KR, Niedobitek G (2008). Integrin alphavbeta6 is a marker of the progression of biliary and portal liver fibrosis and a novel target for antifibrotic therapies.. J Hepatol..

[60] Munoz-Luque J, Ros J, Fernandez-Varo G, Tugues S, Morales-Ruiz M, Alvarez CE (2008). Regression of fibrosis after chronic stimulation of cannabinoid CB2 receptor in cirrhotic rats.. J Pharmacol Exp Ther..

[61] Arrigo T, Chirico V, Salpietro V, Munafo C, Ferrau V, Gitto E (2013). High-mobility group protein B1: a new biomarker of metabolic syndrome in obese children.. Eur J Endocrinol..

[62] Fallowfield JA (2011). Therapeutic targets in liver fibrosis.. Am J Physiol Gastrointest Liver Physiol..

[63] Salpietro C, Cuppari C, Grasso L, Tosca MA, Miraglia Del Giudice M, La Rosa M (2013). Nasal high-mobility group box-1 protein in children with allergic rhinitis.. Int Arch Allergy Immunol..

[64] Chen W, Oue T, Ueno T, Uehara S, Usui N, Fukuzawa M (2013). Apelin is a marker of the progression of liver fibrosis and portal hypertension in patients with biliary atresia.. Pediatr Surg Int..

[65] Meyer DH, Bachem MG, Gressner AM (1990). Modulation of hepatic lipocyte proteoglycan synthesis and proliferation by Kupffer cell-derived transforming growth factors type beta 1 and type alpha.. Biochem Biophys Res Commun..

[66] Leeming DJ, Karsdal MA, Byrjalsen I, Bendtsen F, Trebicka J, Nielsen MJ (2013). Novel serological neo-epitope markers of extracellular matrix proteins for the detection of portal hypertension.. Aliment Pharmacol Ther..

[67] Wang D, Lu JG, Wang Q, Du XL, Dong R, Wang P (2012). Increased immunohistochemical expression of YKL-40 in the spleen of patients with portal hypertension.. Braz J Med Biol Res..

[68] Parfieniuk A, Flisiak R (2008). Role of cannabinoids in chronic liver diseases.. World J Gastroenterol..

[69] Rodriguez-Vilarrupla A, Lavina B, Garcia-Caldero H, Russo L, Rosado E, Roglans N (2012). PPARalpha activation improves endothelial dysfunction and reduces fibrosis and portal pressure in cirrhotic rats.. J Hepatol..

[70] Ryan P, Berenguer J, Michelaud D, Miralles P, Bellon JM, Alvarez E (2009). Insulin resistance is associated with advanced liver fibrosis and high body mass index in HIV/HCV-coinfected patients.. J Acquir Immune Defic Syndr..

[71] d'Annunzio G, Vanelli M, Pistorio A, Minuto N, Bergamino L, Lafusco D (2009). Insulin resistance and secretion indexes in healthy Italian children and adolescents: a multicentre study.. Acta Biomed..

[72] Aleffi S, Petrai I, Bertolani C, Parola M, Colombatto S, Novo E (2005). Upregulation of proinflammatory and proangiogenic cytokines by leptin in human hepatic stellate cells.. Hepatology..

[73] Arrigo T, Gitto E, Ferrau V, Munafo C, Alibrandi A, Marseglia GL (2012). Effect of weight reduction on leptin, total ghrelin and obestatin concentrations in prepubertal children.. J Biol Regul Homeost Agents..

[74] Chirico V, Cannavo S, Lacquaniti A, Salpietro V, Mandolfino M, Romeo PD (2013). Prolactin in obese children: a bridge between inflammation and metabolic-endocrine dysfunction.. Clin Endocrinol (Oxf)..

[75] Mami C, Manganaro R, Marseglia L, Saitta G, Gemelli M, Martino F (2006). Plasma leptin, insulin, and neuropeptide Y response to feeding in newborn infants.. Arch Dis Child Fetal Neonatal Ed..

[76] Delgado MG, Gracia-Sancho J, Marrone G, Rodriguez-Vilarrupla A, Deulofeu R, Abraldes JG (2013). Leptin receptor blockade reduces intrahepatic vascular resistance and portal pressure in an experimental model of rat liver cirrhosis.. Am J Physiol Gastrointest Liver Physiol..

[77] Eslam M, Ampuero J, Jover M, Abd-Elhalim H, Rincon D, Shatat M (2013). Predicting portal hypertension and variceal bleeding using non-invasive measurements of metabolic variables.. Ann Hepatol..

[78] Fallowfield JA, Hayden AL, Snowdon VK, Aucott RL, Stutchfield BM, Mole DJ (2014). Relaxin modulates human and rat hepatic myofibroblast function and ameliorates portal hypertension in vivo.. Hepatology..

